# Targeting Glioblastoma Stem Cells via EphA2: Structural Insights into the RNA Aptamer A40s for Precision Therapy

**DOI:** 10.1021/acs.jcim.5c00295

**Published:** 2025-05-23

**Authors:** Isidora Diakogiannaki, Vincenzo Maria D’Amore, Alessandra Affinito, Greta Donati, Elpidio Cinquegrana, Cristina Quintavalle, Martina Mascolo, Jule Walter, Heike Betat, Mario Mörl, Francesco Saverio Di Leva, Gerolama Condorelli, Luciana Marinelli

**Affiliations:** † Department of Pharmacy, 9307 University of Naples Federico II, Via Domenico Montesano 49, 80131 Naples, Italy; ‡ Institute of Experimental Institute of Endotypes in Oncology, Metabolism and Immunology “G. Salvatore” (IEOMI), Consiglio Nazionale delle Ricerche (CNR), 80131 Naples, Italy; § Department of Molecular Medicine and Medical Biotechnology, University of Naples Federico II, Via Sergio Pansini 5, 80131 Naples, Italy; ∥ Institute for Biochemistry, 9180 Leipzig University, Brüderstraße 34, 04103 Leipzig, Germany

## Abstract

EphA2 receptor tyrosine kinase is overexpressed in many solid tumors and serves as a key driver of tumorigenesis and metastasis. It is highly expressed in glioblastoma multiforme, the most aggressive brain tumor in adults, and in its stem cells [glioblastoma stem cells (GSCs)], which contribute to treatment resistance and tumor relapse. In a previous study, we used the Systematic Evolution of Ligands by Exponential Enrichment (SELEX) procedure, a method for selecting high-affinity nucleic acids to specific targets via iterative selection and amplification, to identify the 2′-fluorinated EphA2-targeting RNA aptamer A40L and a truncated 30-mer derivative, A40s. Both aptamers were able to inhibit GSC growth, stemness, and migration upon EphA2 binding. Here, by integrating computational and experimental methods, the A40s structure was unraveled and its interaction with EphA2 was investigated. Our model offers a blueprint to accelerate the development of optimized A40s variants, advancing next-generation EphA2-targeted anticancer therapies.

## Graphical abstract

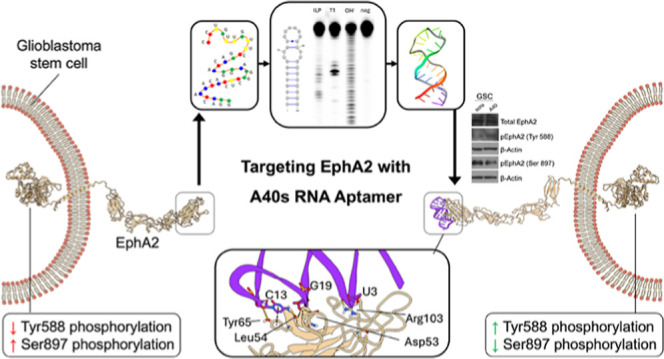

## Introduction

Glioblastoma multiforme (GBM) is the most common and aggressive brain tumor in humans, with a median survival of approximately 14.6 months and a dismal prognosis. Current treatment approaches typically involve tumor surgical resection, whenever feasible, followed by concomitant chemoradiotherapy with temozolomide.^1^ Despite initial improvements in survival with this treatment regimen, tumor progression and relapse are frequent, primarily due to the therapy resistance exhibited by a subpopulation of cells known as glioblastoma stem cells (GSCs), which survive longer than ordinary cells and demonstrate an enhanced ability to withstand conventional therapies, contributing to poor treatment outcomes.^2–4^

Several key proteins are implicated in the pathogenesis of GBM and are associated with tumor proliferation, invasion, and angiogenesis, including epidermal growth factor receptor,^5,6^ vascular endothelial growth factor,^7,8^ platelet-derived growth factor receptor,^5^ and erythropoietin-producing hepatocellular carcinoma A2 (EphA2).^9^ The latter is an elongated transmembrane protein that belongs to the receptor tyrosine kinase family. The extracellular region (ectodomain) consists of an N-terminal ligand-binding domain (LBD), an adjacent cysteine-rich domain, and two fibronectin type III domains (nFN3 and cFN3). The EphA2 ectodomain is connected by a single transmembrane α-helix, which is extended intracellularly to a juxtamembrane region that tethers a tyrosine kinase domain (Figure 1A).

**Figure 1:**
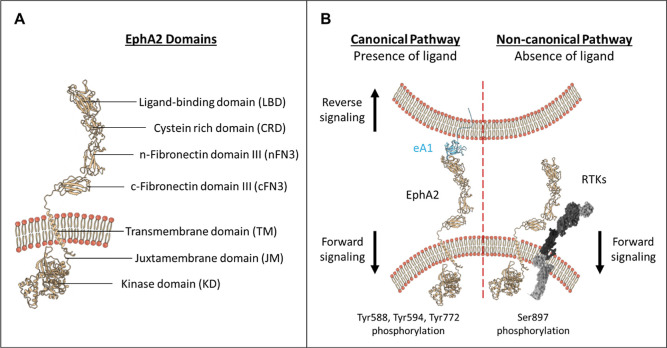
(A) Structural organization of EphA2. (B) Canonical and noncanonical pathways of EphA2. During the canonical pathway, eA1 binds to EphA2, enabling signaling through the receptor EphA2 (forward signaling) and the ligand eA1 (reverse signaling). Upon activation by eA1, EphA2 oligomerizes and provokes autophosphorylation of tyrosine residues in the intracellular domain. In contrast, in the noncanonical pathway, EphA2 heterodimerizes with other RTKs, leading to phosphorylation of Ser897.

EphA2 plays a significant role in various physiological processes, such as cell development, migration, and tissue organization via activation of the so-called canonical pathway.^10^ In this pathway, the ligand ephrinA1 (eA1) binds to the EphA2 LBD, resulting in the oligomerization of EphA2/eA1 complexes and in the following autophosphorylation of Tyr588, Tyr594, and Tyr772 residues within the intracellular domain of the EphA2 receptor. This autophosphorylation facilitates signaling through the receptor (forward signaling) and through the ligand (reverse signaling). In contrast, in the absence of eA1, EphA2 interacts with other proteins such as E-cadherin and integrins, leading to the phosphorylation of Ser897 in EphA2’s intracellular domain, which activates noncanonical signaling.^10–12^ Notably, this noncanonical signaling is associated with tumorigenesis and resistance to therapy (Figure 1B).^10,11,13–16^

Importantly, EphA2 is overexpressed not only in various human malignancies including melanoma, lung cancer, breast cancer, ovarian cancer, and GBM,^12,17–19^ but also in GSCs, allowing these cells to invade the brain more effectively and maintain their stem cell-like properties.^17,18,20^ The overexpression in GSCs contributes to the aggressive behavior of GBM and promotes resistance to conventional therapies. Consequently, EphA2 has emerged as a promising therapeutic target to overcome chemoresistance and improve treatment outcomes in GBM patients. Several strategies have been developed to inhibit this protein, aiming to suppress tumor progression and enhance the efficacy of the existing therapies. For instance, restoring the signaling balance by treating GSCs with EphA2-targeting siRNA^21^ and eA1-Fc (a soluble ephrinA1 dimer fused to Fc)^18^ induces astroglial differentiation, limiting the stem-like properties of the cells and significantly suppressing the growth of xenograft tumors in vivo. Small molecules such as dasatinib have shown potential in pancreatic cancer by inhibiting the EphA2 receptor, disrupting its phosphorylation and key cellular processes.^22^ More importantly, in GBM, dasatinib inhibits critical pathways that drive tumor growth and invasion: preclinical studies demonstrated its strong potential in slowing GBM progression, making it a promising therapeutic option for this aggressive cancer.^23^ Tandem CAR-T cell therapy (TanCAR) destroys glioblastoma tumor cells by recognizing EphA2 and IL-13 receptor α2 in vitro.^24^ However, these strategies face significant limitations due to the lack of selectivity and consequent toxicity, delivery issues, including blood–brain barrier (BBB) membrane permeability, and susceptibility to nucleases.^25–27^

Given these limitations, we adopted an alternative approach to target EphA2 utilizing RNA aptamers due to their potential advantages.^28^ RNA aptamers are short artificial single-stranded RNA sequences that fold into a plethora of heterogeneous three-dimensional shapes, allowing them to form high-affinity, high-specificity binding pockets for their targets. Moreover, by incorporating 2′-fluoro-modified ribonucleotides (2′F-RNA), it is possible to enhance nuclease resistance and improve stability in biological environments.^29,30^ Prior studies have shown that 2′F-RNA preserves local geometry and stacking interactions with only minor perturbations with respect to their nonfluorinated analogues, particularly in short RNAs. Finally, aptamers are easy to synthesize and modify.^31^ The generation of aptamers that target specific cell surface-binding biomarkers is possible using the combinatorial systematic evolution of ligand by exponential [Systematic Evolution of Ligands by Exponential Enrichment (SELEX)] enrichment procedure, a technique used to identify high-affinity nucleic acids that bind specific target molecules through repeated cycles of selection and amplification.^32,33^

Thus, using the latter method, we recently developed the 2′-fluorinated EphA2-targeting RNA aptamer A40L and its truncated 30-mer derivative A40s (5′-ccuGuuGuucGAcAGGAGGcucAcAAcAGG-3′; lowercase letters indicate 2′-fluorinated ribonucleotides).^34^ A40s was demonstrated to interact with EphA2 with high affinity (*K*_D_ = 0.76 ± 0.26 nM) as well as to bind GSCs by EphA2 recognition and inhibit their growth, stemness, and migration. It has exhibited stability in serum in in vitro experiments as well as the ability to cross the BBB in vivo after its systemic injection in healthy mice.^35^

In this study, for the first time, we unveil the three-dimensional structure of A40s and its complex with EphA2, by integrating advanced computational methods with experimental data. Based on prior evidence that 2′-fluoro modifications do not substantially alter the secondary or tertiary structure of short RNAs,^29,30^ computational modeling was conducted using the nonfluorinated version of the aptamer. Notably, our results indicate that A40s interacts with the LBD of EphA2 similarly to the eA1 agonist, suggesting that the aptamer mimics the binding of the endogenous ligand and may restore EphA2 signaling. Importantly, the model allowed us to identify key interaction points critical for binding, laying the foundation for further aptamer optimization aimed at enhancing its binding affinity and therapeutic potential.

## Methods

### Aptamer 2D Structure Prediction

The primary sequence of A40s was used to predict its secondary (2D) structure. To this aim, 11 different online tools were employed: RNAstructure 6.5,^36^ RNAfold 2.6.3,^37^ CentroidFold 0.0.16,^38^ NUPACK 4.0,^39^ Vfold2D 2.5,^40^ Ipknot++ 2.2.1,^41^ MC-Fold 8,^42^ KineFold 3,^43^ pKiss 2.3.0,^44^ sFold 2.2,^45^ and AlphaFold 3.^46^ These servers are based on at least one of the following methods: (1) minimum free energy, which predicts the most probable structure within a folding ensemble (RNAstructure, RNAfold, NUPACK); (2) probabilistic approaches, which use statistical models to provide probabilities for base-pairing interactions (CentroidFold, sFold); (3) experimental data integration, which incorporates experimental data to guide predictions (RNAstructure, Vfold2D); (4) coarse-grained approach that simplifies the RNA structure for computational efficiency (Vfold2D); (5) dynamic folding and kinetics, which simulate the RNA folding process over time, offering insights into folding pathways and kinetics (Kinefold); (6) pseudoknots handling, which are specifically designed to predict RNA structures with pseudoknots (IPknot++, McFold, KineFold, pKiss); and (7) machine learning-based probabilistic approaches, which apply deep learning algorithms trained on large data sets to predict complex structures with high accuracy (AlphaFold 3). All available parameter settings provided by each tool were considered for the 2D structure prediction. For AlphaFold 3, which allowed for both isolated RNA and RNA–protein complex predictions, we included 2D structures of A40s predicted alone as well as those predicted in complex with EphA2. In cases in which multiple structures with varying free energies were obtained, we selected the top three solutions, resulting in a total of 46 predicted 2D structures. These structures were clustered with in-house Python scripts that employed affinity propagation (Figure S1), and then analyzed based on the results of the probing experiments.

### In Silico 3D Structure Prediction of A40s

For the tertiary structure prediction of the two selected 2D configurations, hairpin (dot bracket “((((((((...........))))))))”) and bulged (dot-bracket “((((((((..((.......))))))))))”) (details provided in the Results section), the aptamer’s sequence and dot-bracket notation were used as input. Structure predictions were performed by using three well-established programs for RNA 3D predictions: RNAComposer,^47^ 3dRNA,^48^ and Vfold3D,^49^ all with default parameters. For each of the two predicted 2D structures, the top three scored conformations from RNAComposer and 3dRNA were selected. Additionally, a single conformation generated by Vfold3D was used for the hairpin structure, while two conformations, being the only ones produced by Vfold3D, were used for the bulged structure. A total of seven hairpin and eight bulged 3D conformations were identified. To analyze structural diversity, the conformations from the two models were clustered separately using two complementary strategies: (i) clustering based on backbone root-mean-square deviation (RMSD), applying the GROMOS^50^ algorithm with a 4.5 Å cutoff, and (ii) clustering based on the epsilon RMSD (eRMSD) metric through the Barnaba software.^51^ For eRMSD-based clustering, G-vectors were first computed from the aptamer conformations to capture base-level structural features. These vectors were then projected onto the first two principal components derived from a principal component analysis, and clustering was performed using the DBSCAN algorithm by setting *min_samples* = 2 and *eps* = 0.9. Setting the minimum number of samples to 2 was essential due to the limited size of the data set. Notably, smaller *eps* values led Barnaba to fail in detecting any clusters, which justified the chosen parameters. Both clustering approaches converged on the same representative conformation from the most populated cluster of each model. These structures were selected for subsequent molecular dynamics (MD) simulations.

### System Setup and Atomistic MD Simulations of A40s Models

All simulations were performed on the nonfluorinated RNA sequence of A40s. In fact, the 2′-fluoro-modifications are not expected to significantly affect the aptamer’s secondary or tertiary structure, as supported by the prior literature.^29,30^ Three replicas of each system, namely hairpin and bulged, were simulated for 3 μs using the GROMACS[Bibr ref52] 2020.6 software. For the replica simulations, all conditions were identical apart from different initial velocities. The leap program available in AmberTools^53^ was used to prepare each system for the MD simulations. The aptamer was described using the OL3 ^54^ force field and then solvated in a TIP3P^55^ water cubic box with a 12.0 Å edge distance under periodic boundary conditions (PBC). 150 mM K^+^ and Cl^–^ ions, modeled with Joung and Cheatham parameters,^55,56^ were randomly placed to neutralize the total charge of the system and mimic physiological conditions. Finally, coordinates and topology files for all of the systems were obtained.

The Verlet cutoff scheme was used for nonbonded interactions neighbor search, whereas the long-range electrostatic interactions were treated using the particle-mesh Ewald summation method (PME).^57^ Short-range Coulombic and van der Waals interactions were treated with a cutoff distance of 12 Å.

Prior to MD simulations, all structures were relaxed with energy minimization, followed by subsequent NVT and NPT runs. The energy minimization consisted of two steps performed by using the steepest descent algorithm: (i) 20,000 steps, with harmonic restraints of 5000 kJ mol^–1^ nm^–2^ applied to the aptamer heavy atoms, so that only the solvent was unconstrained; and (ii) 50,000 steps during which the entire system was allowed to relax.

To integrate Newton’s equations of motion, the leapfrog^58^ algorithm was employed and a time step of 2 fs was chosen, while the LINCS^59^ algorithm was used to constrain bonds involving hydrogen atoms. During the MD equilibration procedure, the system was gradually heated by increasing the temperature with subsequent MD runs in the canonical ensemble (NVT) using the weak-coupling Berendsen^60^ scheme. Specifically, three 500 ps NVT steps were performed by gradually increasing the temperature from 100 K up to 300 K. At each step, harmonic restraints were applied to all heavy atoms of the aptamer and were gradually decreased from 1000 to 500 and finally to 250 kJ mol^–1^ nm^–2^. Subsequently, two NPT equilibration runs of 1 and 5 ns, respectively, were performed to adjust the box volume using the Berendsen algorithm for pressure coupling. In the first NPT step, restraints of 100 kJ mol^–1^ nm^–2^ were applied on heavy atoms, while no restraints were used in the last equilibration run.

During the 3 μs production runs, the temperature was kept constant at 300 K using the v-rescale^61^ thermostat with a time constant of 0.5 ps. The pressure was isotropically maintained at 1 atm using the Parrinello–Rahman^62^ barostat with a time constant of 5 ps and a compressibility of 4.5 × 10^–5^ bar.

For each aptamer configuration, the RMSD and root-mean-square fluctuations (RMSF) were analyzed along with the stability and formation of hydrogen bonds throughout the trajectory. For the hairpin configuration, which showed the highest agreement with experimental data, all frames from the three replicas were clustered using the GROMOS algorithm with a 4.5 Å cutoff. A representative conformation was then selected from the most populated cluster.

### In-Line Probing of In Vitro Transcribed A40L Aptamer

T7-based in vitro transcription was used to generate the A40L aptamer. The resulting RNA was purified via denaturing 10% polyacrylamide gel electrophoresis and subsequent ethanol precipitation. The aptamer transcript was incubated with 5 U Antarctic phosphatase (NEB) for 1 h at 37 °C to dephosphorylate the 5′-end. Radioactive 5′-end labeling was performed using 10 μCi γ-^32^P-ATP (Hartmann Analytic), 8 mM DTT, 1× PNK buffer, and 20 U T4 polynucleotide kinase (NEB). The reaction was incubated at 37 °C for 1 h, and the reaction product was purified via a second denaturing polyacrylamide gel electrophoresis. 10 pmol of the labeled aptamer RNA (corresponding to 30,000 cpm) were denatured for 1 min at 90 °C and cooled down to 37 °C for 5 min for refolding. ILP buffer was added to a final concentration of 50 mM Tris/HCl pH 8.5, 20 mM MgCl_2_, and 100 mM KCl. Structure analysis was performed according to Regulski and Breaker (2008) with slight variations.^63^ To this end, the RNA was incubated for 40 h at room temperature. Untreated RNA served as a negative control. As size standards, aliquots of the labeled transcript were incubated for 2 min at 37 °C with 0.05 U RNase T1 (resulting in a G-specific cleavage pattern at single-stranded positions) and in the presence of 50 mM Na_2_CO_3_/NaHCO_3_ (5 min at 95 °C), resulting in partial alkaline hydrolysis. Reactions were stopped by the addition of 3× colorless loading dye and 13.5 mM EDTA. Reaction products were separated on a denaturing 8% polyacrylamide gel and visualized using a Typhoon FLA 9410 (GE) PhosphorImager.

### Cell Lines

Patient-derived GSCs^64^ were provided by Dr. Lucia Ricci Vitiani’s lab and cultured as previously reported.^34,35^

### Western Blot Analysis

GSCs were seeded in 12-well plates and treated with 400 nM either A40s or a scrambled aptamer as a negative control. EphA2-receptor phosphorylation was evaluated in GSCs after 24 h of treatment. Phosphorylation of RSK2 and AKT was analyzed following a 3 h cell starvation in BTSC medium^64^ depleted of EGF and FGF and diluted 1:1 with F12 serum-free medium. Subsequently, cells were stimulated for 1 h with F12 medium supplemented with 10% FBS and treated with 400 nM either A40s or the scrambled control. Western blotting was performed as described previously.^34,35^ The primary antibodies used included EphA2 sc-398832 from Santacruz Biotechnologies (Texas, USA) and phosphor-Akt Ser473 (9271), Akt pan (4685), phosphor-EphA2 Tyr588 (12677), and Phospho RSK2Ser227 (D53A11) from Cell Signaling Technology (Milan, Italy). Bands were quantified using an ImageJ Gel Analyzer, and the calculated values were expressed as fold change relative to the scrambled control. The effects of A40s on phosphorylated proteins were determined relative to the respective corresponding total protein levels, following prior normalization of each sample to its own β-actin quantity.

### RNA-Protein Docking

To obtain a model of EphA2 bound to the aptamer, we utilized HADDOCK 2.4 ^65^ and ZDOCK 3.0.2 ^66^ web servers for protein-nucleic acid docking. The MD-predicted aptamer hairpin conformation was selected for docking calculations. The atomistic model for EphA2 was retrieved from the Protein Data Bank (PDB) (PDB ID: 3FL7),^67^ which includes the entire extracellular domain of EphA2 in the apo form. The LBD was isolated and prepared using the Protein Preparation Wizard tool (Schrödinger 2022-4).^68^ During this step, missing residues were added and refined using Prime,^69,70^ tautomer/ionization states were assigned, and the final structures were minimized. Surface residues of the LBD were selected as active residues for rigid docking using default parameters. Ultimately, the structure with the lowest energy from each software suite was selected for the subsequent MD simulations.

### Setup and MD Simulations of EphA2/A40s and EphA2/eA1 Complexes

The EphA2/A40s complex derived from molecular docking was selected for MD simulations, while the atomistic model for EphA2/eA1 was retrieved from the PDB ID: 3CZU, which includes the LBD of EphA2 bound to its endogenous ligand, eA1.

The systems were prepared using the leap program available in AmberTools.^53^ The ff14SB^71^ and OL3 ^54^ force fields were used to describe the proteins and the aptamer, respectively. Each complex was solvated in a TIP3P^55^ water cubic box with a 15.0 Å edge distance under PBC. To neutralize the total charge of the EphA2/A40s system and mimic physiological conditions, 33 Na^+^ ions, modeled with Joung and Cheatham parameters,^55,56^ were added. Finally, coordinates and topology files for the two systems were generated. The equilibration and MD production protocols followed were the same as those previously described. Each complex was simulated for 5 μs using the GROMACS 2020.6 software.^52^

Trajectories visualization, RMSD, and time series of bonds between nucleotides analyses were performed with the VMD^72^ software. The RMSF calculations and cluster analysis on the MD trajectory of the aptamer were carried out using specific tools implemented in GROMACS.^52^ eRMSD analysis was performed with Barnaba.^51^

## Results

### In Silico 2D Structure Prediction of A40s

To elucidate the 2D structure of A40s, the following web servers were utilized: RNAstructure,^36^ RNAfold,^37^ CentroidFold,^38^ NUPACK,^39^ Vfold2D,^40^ Ipknot++,^41^ MC-Fold,^42^ KineFold,^43^ pKiss,^44^ sFold,^45^ and AlphaFold3.^46^ These servers allowed us to capture a broad spectrum of 2D predictions, generating a total of 46 models, which were then clustered based on their structural similarity. The most populated cluster (13 out of 46 predictions) represented a hairpin structure, consisting of an 8-base-paired stem and a 14-nucleotide loop (dot-bracket notation: “((((((((...........))))))))”) or a 9-base paired stem and a 12-nucleotide loop (dot-bracket notation: “(((((((((..........)))))))))”), while the second most populated cluster (10 out of 46 structures) represented a left-bulged hairpin model (bulged), including an 8-base paired stem, a 2-nucleotide bulge, two base pairs between G11-C22 and A12-U21, and an 8-nucleotide loop (dot-bracket notation: “((((((((..((.......))))))))))”) (Figure 2). A detailed table listing the employed tools and their corresponding solutions is provided in the Supporting Information (Figure S1).

**Figure 2:**
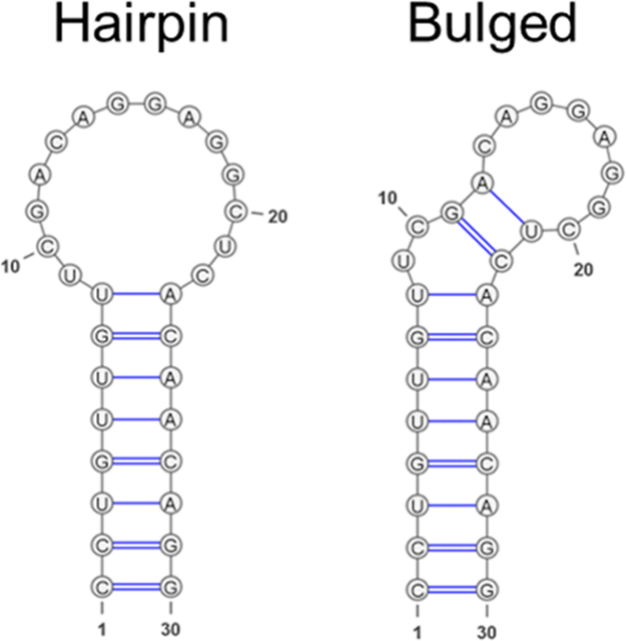
Hairpin and hairpin-bulged (bulged) models derived from clustering of 2D RNA structures. The 2D RNA representations shown were generated using VARNA.^73^

### In Silico 3D Structure Prediction of A40s

For the tertiary structure prediction of the hairpin and bulged models, we used three well-established programs for RNA 3D predictions: RNAcomposer,^47^ 3dRNA,^48^ and Vfold3D.^49^ The accuracy of these methods has been validated in previous studies on five aptamers, with all of them showing high accuracies for the 3D structure prediction of short aptamers (<40 nucleotides).^74^ The use of multiple tools allowed us to explore a variety of conformations and evaluate the convergence. Indeed, seven and eight possible 3D conformations were obtained for the hairpin and bulged models, respectively, which were then clustered by using a cutoff of 4.5 Å. Regarding the hairpin model, all conformations were grouped into a single, cohesive cluster characterized by substantial overlap in both the stem and loop regions. In contrast, the bulged model yielded three distinct clusters with the most populated cluster accounting for 50% of the sampled conformations. While the conformations within this dominant cluster exhibited consistent alignment in the stem region, variations were observed in the orientation of the unpaired bases, reflecting structural heterogeneity in the bulge region (Figure 3). To further validate these observations, we performed an independent cluster analysis using the nucleic acid-specific eRMSD metric.^75^ Unlike RMSD, eRMSD focuses solely on the relative positions and orientations of nucleobases, making it particularly sensitive to base-pairing and base-stacking interactions. Consequently, it offers a complementary perspective to backbone-oriented RMSD-based clustering. Interestingly, the centroids identified through eRMSD closely matched those obtained with RMSD clustering. However, eRMSD classified a larger proportion of structures as outliers (Figure S3A), likely due to its heightened sensitivity to local base orientation and the relatively small sample size. Moreover, while RMSD analysis suggested higher structural conservation within the hairpin ensemble, eRMSD-based clustering revealed reduced variability among bulged structures (Figure S3B). This apparent discrepancy can be explained by the distinct features of the two predicted motifs, particularly a different number of base pairs involving nucleotides 9–22, which are emphasized by the two metrics. The hairpin structures contain no base pairs in this region and, in turn, a more flexible loop, which may adopt diverse base orientations across predictions, leading to greater variation in eRMSD. In contrast, bulged structures, although more variable in backbone geometry, tend to maintain more consistent local base orientation due to two additional base pairs involving nucleotides G11-C22 and A12-U21. This interpretation is further supported by cross-eRMSD matrices computed for all hairpin and bulged structures, excluding the loop region (Figure S3C). When this loop is excluded, the analysis aligns more closely with the RMSD-based results (Figure S3D,E), suggesting that loop flexibility is a major contributor to the divergence among 3D predictions, an aspect that warrants further investigation in a dynamic context.

**Figure 3:**
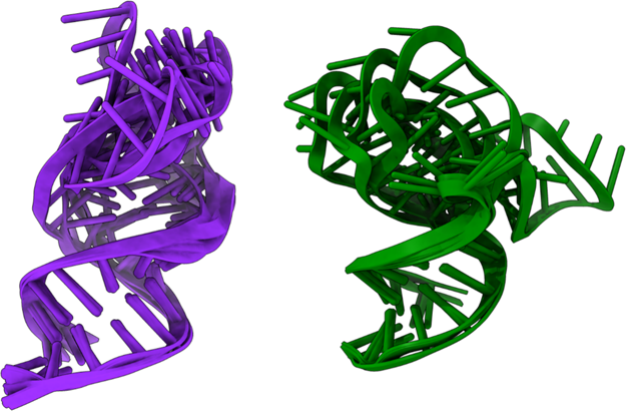
Ensemble of 3D conformations of the hairpin structure (left, violet cartoon) and bulged structure (right, dark green cartoon) obtained using RNAcomposer,^46^ 3dRNA,^47^ and Vfold3D.^48^

### Molecular Dynamics of A40s in Solution

The stability and energetics of the A40s hairpin and bulged predicted models were then investigated through 3 μs full-atom MD simulations in three replicas using GROMACS 2020.6 ^52^ with the OL3 ^54^ force field. RMSD calculation of the aptamer’s heavy atoms (Figure 4A) and eRMSD (Figure 4C) with respect to the initial frame confirmed the general stability of the hairpin conformation (averaged RMSD_hairpin_ = 2.8 ± 0.4 Å, averaged eRMSD = 0.85). Subsequent RMSF analysis of the hairpin structure indicated flexibility in the terminal nucleotides, nucleotides 13 and 15–19 across all replicas, and nucleotide 9 only in the first replica, with RMSF values reaching 2.5–3.0 Å (Figure 4E). Notably, all of these nucleotides are part of the hairpin loop. Monitoring hydrogen bonds between the bases of the hairpin model revealed the noteworthy formation of a new base pair between nucleotides G11 and C20, which was consistently present in all three replicas (93.1%, 96.0%, and 91.6%, respectively). Additionally, the formation of a noncanonical base pair between nucleotides A12 and G19 was observed in all three replicas, with frequencies of occurrence of 52.6%, 51.5%, and 49.7%, respectively, which indicates transitioning between paired and unpaired bases (Figure 5A). Compared to the hairpin model, the bulged model demonstrated reduced stability across all simulations, as reflected by the RMSD value of the aptamer’s heavy atoms, measured at 4.6 ± 1.2 Å (Figure 4B), and an average eRMSD of 1.12 (Figure 4D) across the three replicas. In line with this observation, the RMSF values for the terminal and unpaired nucleotides (10 and 16–18) ranged from 4.5 to 7.0 Å (Figure 4F), while the expected paired nucleotides (11–12 and 20–22) exhibited lower values between 4.0 and 5.0 Å (Figure 4F). This structural instability is further emphasized by pronounced changes in the aptamer’s 2D arrangement compared to its initial configuration. As shown in Figure 5A, the three simulations failed to converge to a single conformation, underscoring the low thermodynamic stability of the initial bulged structure. For the sake of thoroughness, we also evaluated the potential overlap in conformational space between the two sets of RNA-only simulations. For each simulated replica, we computed the cross-eRMSD relative to the hairpin structure (for simulations initialized from the bulged conformation) and the bulged structure (for those initialized from the hairpin conformation). As shown in Figure S5, the resulting plots indicate that no interconversion occurs between the two conformations in any of the replicas over the 3 μs simulation time.

**Figure 4:**
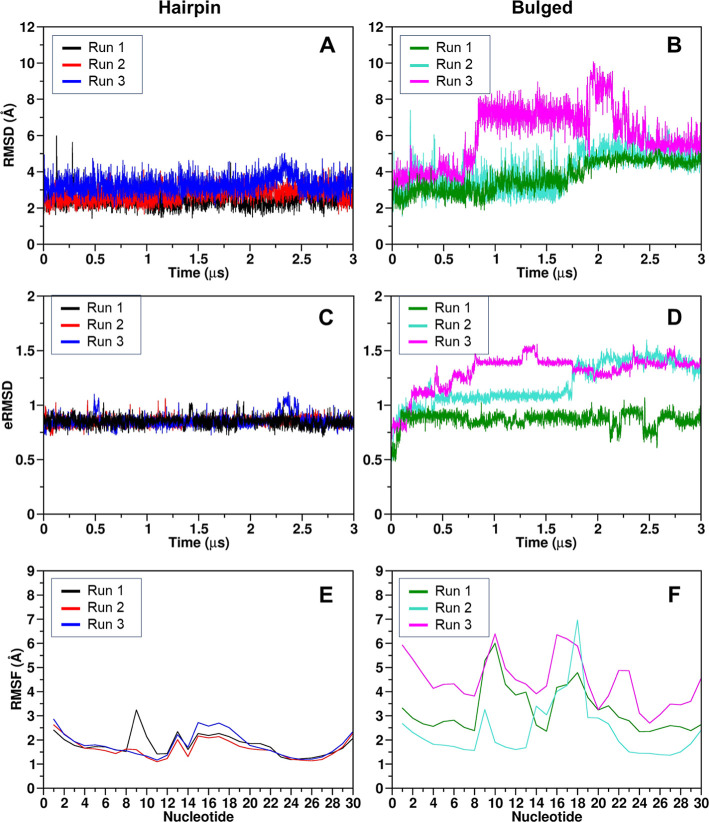
RMSD (top panels), eRMSD (central panels), and RMSF (bottom panels) plots of the hairpin (A,C,E) and bulged (B,D,F) models. The values are computed on the A40s’s heavy atoms with respect to the initial frame. For the RMSD calculations, the terminal nucleotides were excluded.

**Figure 5:**
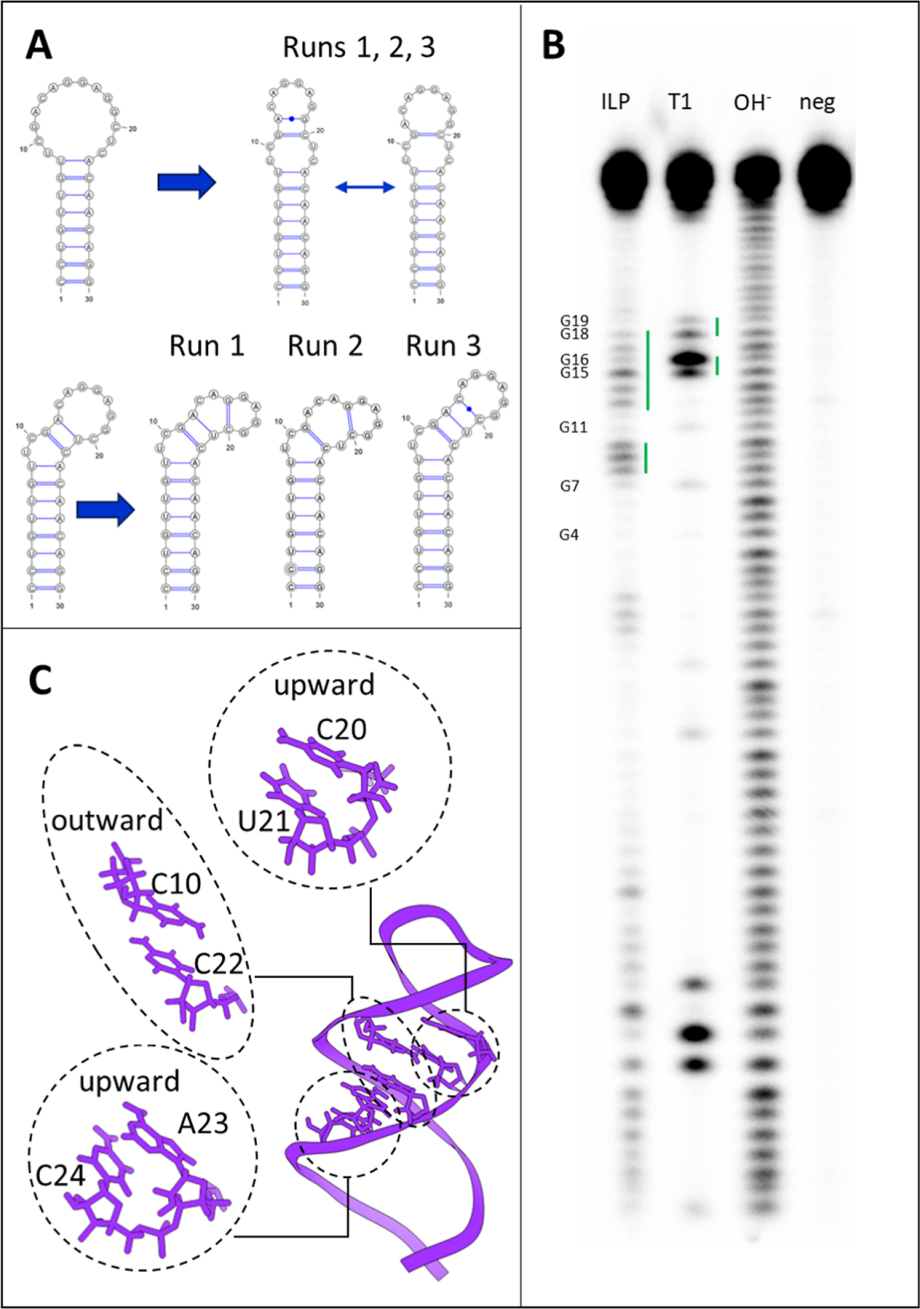
(A) Changes of the A40s’s secondary structures during MD simulations for both the hairpin and the bulged conformation. (B) Structure probing results on the full length of the aptamer (A40L), where signals indicate unpaired nucleotides. Unpaired nucleotides (labeled according to the corresponding position in the shorter aptamer’s sequence) are highlighted with green lines. (C) Base stacking interactions in the hairpin model.

This observation suggests that the transition between these structural states likely occurs on time scales longer than those sampled here, implying a lower bound for the interconversion rate underequilibrium conditions. Notably, replicas initiated from the same conformation tend to converge toward similar cross-eRMSD values, further supporting consistent sampling within each system.

### Validation of the Predicted A40s Structure Using Probing Experiments

To validate the computational predictions, we conducted ILP and RNase T1 cleavage experiments according to Regulski and Breaker.^63^ ILP results in a preferential cleavage of single-stranded positions and thus offers valuable insights into the 2D structure by revealing band signals at the cleaved sites. While the ILP reaction targets unpaired positions without base specificity, RNase T1 specifically cleaves at guanine residues, with a strong preference for single-stranded regions. To enhance band clarity and simplify the interpretation of the probing results, we used the longer version of the aptamer, A40L,^34^ which provides additional cleavage sites. The extended sequence not only minimizes the risk of ambiguous results but also improves RNA stability during handling, streamlining the experimental workflow.

Figure 5B illustrates the results of the probing experiments, highlighting signals that indicate unpaired nucleotides. ILP data suggest that nucleotides 1–7, 11–12, and 19–30 are paired, while 8–10 and 13–18 are unpaired; RNase T1 cleavage suggests that nucleotides G15, G16, G18, and G19 are unpaired, while G4, G7, and G11 are paired. Altogether, these observations support the MD-predicted A40s conformation. Nonetheless, nucleotide G19 is classified as paired in the ILP lane but seems to be unpaired in the RNase T1 cleavage reaction (Figures 5B and S6). This discrepancy can be explained by the fact that unpaired bases can form stacking interactions with the neighboring bases.^76,77^ If such stacking limits the conformational freedom of G19, in-line-induced cleavage might be strongly reduced. Furthermore, the computational data indicate that G19 transiently forms a noncanonical base pair with A10, with a frequency of occurrence of 50% in the three simulations. Hence, it is possible that such a transient pairing contributes to a partially paired character of G19, as indicated in the ILP analysis.

On the other hand, there is a mismatch between the computational predictions and the experimental data for nucleotides 21–23. Indeed, the computational model predicted these nucleotides to be unpaired, whereas the ILP results showed either very weak signals or no signal at all. Visualization of the A40s conformation revealed potential base-stacking interactions in this region, which could reduce its accessibility for cleavage, resulting in the absence of a signal (Figure 5C). It is worth noting that minor discrepancies between RNase T1 and ILP reactivity, such as at G19 and nucleotides 21–23, likely reflect differences in strand break mechanisms and local RNA dynamics, as previously observed for other structured RNAs.^78^

In contrast, the different conformations observed at the end of the bulged MD simulations do not align with the experimental data. In particular, the paired G15 in replicas 2 and 3 is inconsistent with the RNase T1 cleavage pattern that exhibits a clear, intense band signal indicative of an unpaired nucleotide and the paired C13 in replica 3 that was expected to remain unpaired according to ILP data. Altogether, these results provide strong support for the predicted MD conformation of the aptamer, reinforcing our confidence in the computational hairpin model.

### A40s Modulates EphA2 Phosphorylation and Downstream Signaling in GSCs

We performed Western blot analysis on patient-derived GSCs to evaluate the ability of A40s to modulate the EphA2 canonical and noncanonical signaling pathways. Our observations show that A40s treatment promotes EphA2 phosphorylation at Tyr588, which is associated with canonical EphA2 signaling through its natural ligand eA1 to maintain cellular homeostasis. Concurrently, A40s treatment reduces EphA2 phosphorylation at Ser897,^79^ which is instead linked to noncanonical signaling and receptor’s oncogenic activity^80^ (Figure 6). Furthermore, A40s treatment also decreased the levels of phosphorylated RSK2 and AKT (pAKT) (Figure 6), which are both known to activate the oncogenic function of EphA2. These findings suggest that A40s acts as a functional mimic of the endogenous ligand eA1 and impairs the oncogenic activation of EphA2 receptor, highlighting its potential for GSCs-targeted therapy.

**Figure 6:**
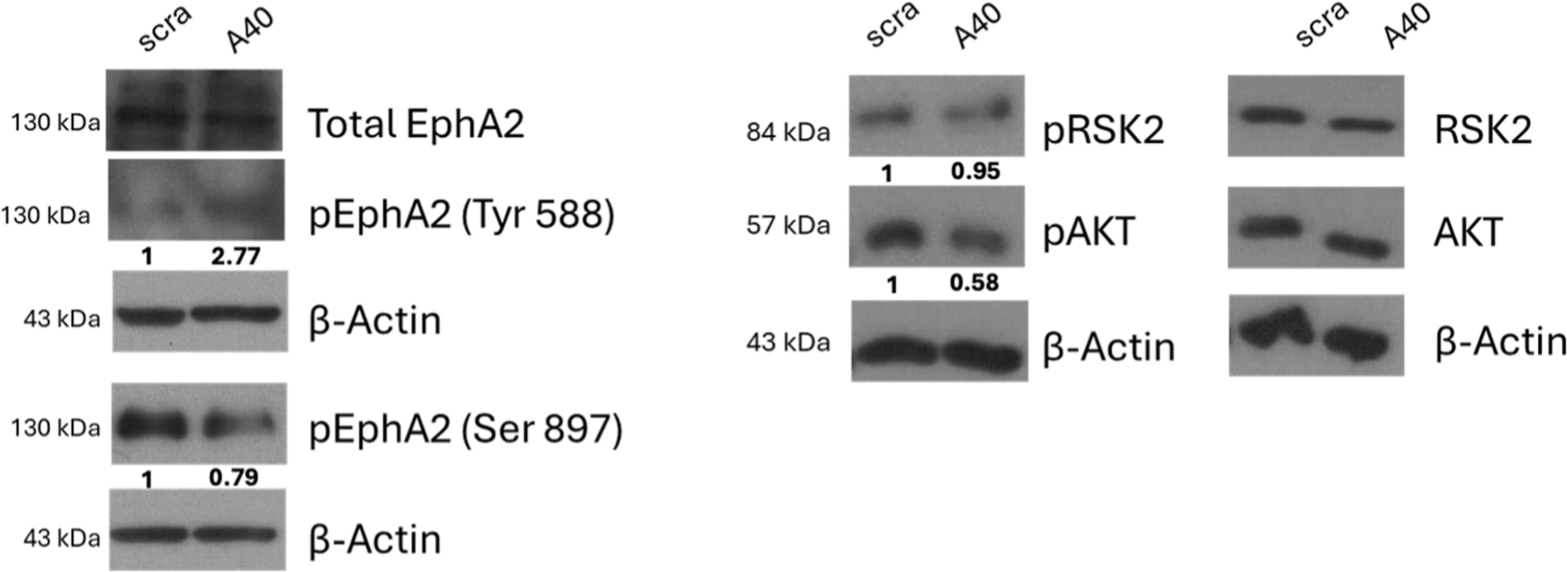
Western blot analysis of phosphorylation of EphA2 (left) and associated kinases RSK2 and AKT (right). Bands were quantified using the ImageJ Gel Analyzer tool. Densitometric values were normalized to β-actin and expressed as fold change relative to the scramble aptamer control. Phosphorylated protein levels (pEphA2 at Tyr588 and Ser897) were normalized to total EphA2 levels in each sample.

### Modeling EphA2/A40s Interaction

To elucidate the binding of A40s to EphA2, we initially performed molecular docking using the HADDOCK 2.4 ^65^ and ZDOCK 3.0.2 ^66^ servers, both widely cited in the literature for predicting nucleic acids–protein interactions.^74,81–87^ HADDOCK requires the selection of up to 150 active residues for docking simulations. As the EphA2 protein has 427 surface residues, we focused our docking efforts exclusively on the LBD. In fact, Western blot data suggest that our aptamer mimics eA1 by inducing phosphorylation patterns consistent with canonical signaling. Given that eA1 binds specifically to the LBD of EphA2, we concentrated our docking analysis on this region. Notably, the lowest-energy poses provided by the two programs converged to the same conformation (RMSD = 0.3 Å), in which A40s adopts a horizontal position on the top of EphA2, with nucleotides 19–22 entering within the surface pocket of the LBD (Figure 7). In addition, we used AlphaFold 3,^46^ an advanced machine learning tool designed to predict RNA–protein complexes using only sequence data as input. AlphaFold successfully predicted the binding of A40s to the surface of EphA2, with the loop inserted into the EphA2 structure (Figure S2). However, it failed to accurately predict the secondary structure of A40s, leading us to exclude this model from further analyses.

**Figure 7:**
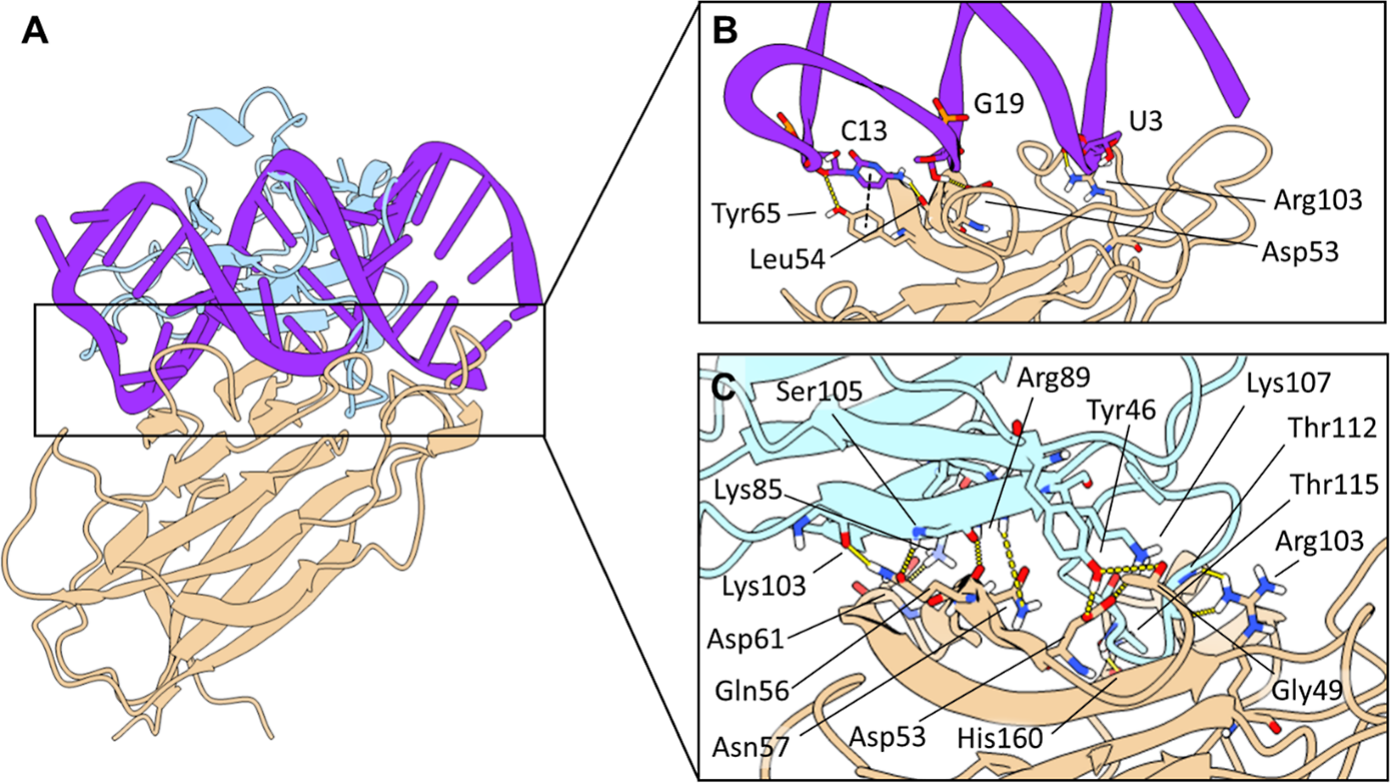
(A) Superposition of the EphA2/A40s complex predicted by HADDOCK 2.4 and the EphA2/eA1 X-ray complex (PDB ID: 3CZU) showing that A40s and the endogenous ligand occupy the same binding surface. Graphical representation of the most important interactions between EphA2 and A40s (B) and between EphA2 and eA1 (C) are shown as insets. The main interacting residues are illustrated as sticks, with specific interactions highlighted by dashed lines. EphA2 is colored in beige, A40s in violet, and eA1 in cyan.

The predicted complexes of the EphA2/A40s by HADDOCK and ZDOCK were aligned with the natural EphA2/eA1 complex (PDB ID: 3CZU),^67^ revealing that A40s and eA1 occupy the same binding region, as illustrated in Figure 7. We then investigated the stability and energetics of the docking-predicted EphA2/A40s complex by performing 5 μs all-atom MD simulations using GROMACS 2020.6 software. Visualization of the trajectory and plotting of the RMSD of A40s relative to the protein revealed a slight shift of the aptamer at approximately 200 ns of the simulation (Figure S7) to reach a new arrangement that is conserved for the rest of the simulations. In addition, the overall conformation of A40s itself displayed enhanced stability with an RMSD value of 2.7 ± 0.3 Å along the whole trajectory, and monitoring the hydrogen bonds between its base pairs indicated the preservation of its 2D structure throughout the 5 μs (Figure S8).

Furthermore, we evaluated structural refinement of the A40s/Eph2 interaction throughout the simulation by computing the DRPScore.^88^ DRPScore is a deep-learning-based scoring tool designed to evaluate the nativity of RNA-protein complexes based on known complexes, taking into account atom positions, residue types, intermolecular distances, and interaction interfaces. As shown in Figure S9, DRPScore increases from an initial value of 0.85 (corresponding to the docking pose) to 0.99 over the simulation, indicating that the final MD model achieves a high degree of native-likeness according to this metric. These results confirm that while docking provides a reasonable initial model, MD refinement is crucial for capturing a more native-like and stable interaction interface that static docking poses might miss. The DRPScore analysis independently validates the structural refinement achieved, adding extra strength to our MD predictions, as the probability of a native-like structure converges to 99.9% across the trajectory.

To investigate the structural basis of EphA2 and A40s binding, we utilized PyContact,^89^ a toolkit designed to analyze noncovalent biomolecular interactions in MD trajectories. Our analysis identified the most significant aptamer–protein interactions, focusing on those that persisted for at least 0.4 μs during the simulation (Figure S10). These interactions involve amino acids Asp53, Leu54, Tyr65, and Arg103 of EphA2, as well as nucleotides C2, U3, C13, and G19. Hydrogen bonds are formed between the carboxylic oxygen of Asp53 and the hydroxyl sugar oxygen of G19, the backbone oxygen of Leu54 and the amino nitrogen of C13, and the hydroxyl oxygen of Tyr65 and the sugar oxygen of C13. Additionally, a salt bridge is formed between the positively charged guanidinium nitrogen of Arg103 and the negatively charged phosphate oxygen of U3. Lastly, a T-shaped π–π interaction is formed between the aromatic phenolic ring of Tyr65 and the pyrimidine ring of C13 (Figure 7B).

In parallel, we performed 5 μs MD of the reference EphA2/eA1 system by utilizing the same protocol. RMSD analysis of secondary structure showed, as expected, high stability of eA1 (the ligand, L) bound to EphA2 (the receptor, R), with a value of 2.8 ± 0.8 Å (Figure S11). Intermolecular interaction analysis revealed hydrogen bonds between the backbone oxygen of ^R^Gly49 and the hydroxyl group of ^L^Tyr46 and between the latter and the ^R^Asp53 carboxyl moiety. Additional H-bonds are formed by the ^L^Arg89 guanidinium group with both the side chain nitrogen of ^R^Asn57 and the backbone oxygen of ^R^Gln56 and between the side chain atoms of the latter with both ^L^Ser105 and ^L^Lys103 backbone atoms. Finally, H-bonds are established between the ^R^Arg103 side chain and the ^L^Thr112 backbone atoms and between the ^R^His160 backbone atoms and the ^L^Thr115 hydroxyl. Additionally, salt bridges are formed between negatively charged ^R^Asp53 and positively charged ^L^Lys107, as well as between ^R^Asp61 and ^L^Lys85 (Figure 7C). Notably, in the crystal structure, the interactions between ^R^Gly49 and ^L^Asp53, ^R^Asn57 and ^L^Arg89, ^R^Asp61 and ^L^Lys85, and ^R^Arg103 and ^L^Thr112 were absent or characterized by a distance of 5.22, 4.30, 4.62, and 3.85 Å (measured between heavy atoms), respectively, which exceeds the typical range for hydrogen bonding or salt bridge. During the MD simulations, these interactions are formed and stabilized, suggesting that dynamic conformational changes or flexibility facilitated their establishment. In contrast, only a single interaction between ^R^Arg103 and ^L^Glu119, observed in the crystal structure, is disrupted or formed for less than 40% of the simulation time. These observations underscore the importance of MD simulations in capturing conformational states and interactions not observed in static crystal structures. Notably, in both EphA2/eA1 and EphA2/A40s, residues Asp53 and Arg103 play pivotal roles in mediating intermolecular interactions. This finding suggests that these residues may serve as conserved interaction sites, potentially underlying a well-defined mechanism of molecular recognition or binding affinity.

## Discussion and Conclusions

GBM is the most aggressive brain tumor in humans, with a poor prognosis largely attributed to the presence of GSCs. Indeed, these cells promote therapy resistance and drive tumor relapse, underscoring the need for more effective therapeutic strategies. EphA2, a protein overexpressed in GSCs, is a promising target for such approaches. Despite extensive efforts to target EphA2 using small molecules, peptide-based approaches, and antibodies, these strategies are hindered by limitations such as lack of specificity, toxicity, and delivery inefficiencies.

In our research pipeline, we followed a novel RNA-based approach utilizing RNA aptamers because of their potential advantages, including high specificity, low toxicity, and low susceptibility to degradation by nucleases, the small size, and the ease of synthesis and modification. After the FDA approval of pegaptanib (Macugen), an RNA aptamer for the treatment of neovascular (wet) age-related macular degeneration,^90^ and avacincaptad pegol (Izervay), a complement inhibitor for the geographic atrophy treatment,^91^ strategies focusing on RNA aptamers hold great promise for targeting a wide range of human diseases.^91,92^

In this study, we focus on A40s, a 30 nt RNA aptamer (5′-ccuGuuGuucGAcAGGAGGcucAcAAcAGG-3′) recently developed by us, which incorporates 2′-fluoro-modified nucleotides to enhance nuclease resistance and improve stability in biological environments, with minimal disruption to local and overall helix geometry.^29,30^ Our aptamer selectively binds to GSCs by EphA2 recognition and effectively inhibits GSCs’ growth, stemness, and migration.^34,35^ These properties address the shortcomings of previous strategies and highlight the potential of A40s as a targeted and effective therapeutic approach for EphA2-positive cancers.

Here, we integrated advanced computational and experimental methods to develop a structural model of A40s as well as of the aptamer bound to its target, EphA2. First, we predicted the secondary and tertiary structure of A40s, which resulted in a hairpin consisting of an 8-base-paired stable stem and a more flexible 14-nucleotide loop. Notably, MD simulations supported this structure, revealing additional base pairs and stacking interactions that were absent in the initial software predictions. These findings were corroborated by ILP and RNase T1 cleavage experiments, providing a reliable A40s model for further investigations of its interaction with EphA2.

This underlines the importance of integrating advanced computational and experimental methods in understanding RNA molecules and biological systems. Simple computational tools built upon different algorithms and prediction strategies offer powerful insights. Furthermore, when combined with advanced theoretical methods, they can reveal crucial details that basic experiments might fail to predict. On the other hand, experimental methods validate predictions and unveil structural information that computational methods alone might overlook. By capitalizing on both computational and experimental strengths, we thus advance our ability to study, understand, and design complex biomolecular structures.

Molecular docking studies between A40s and EphA2 proposed a potential binding mode where A40s aligns horizontally atop EphA2, with nucleotides 19–22 in the aptamer loop engaging the LBD surface pocket. This configuration resembles the experimental binding pose of endogenous ligand eA1, suggesting a shared interaction mechanism. AlphaFold identified a similar binding region, while it was unable to accurately predict the aptamer’s secondary structure, highlighting the limitations of current AI-based tools and the need for further refinement to improve predictive accuracy. On the other hand, MD simulations on the EphA2/A40s complex suggest that the aptamer could undergo structural post-binding adjustments to stabilize into a conformation consistent with its predicted structure. This finding hints at the dynamic nature of RNA–protein interactions and underscores the potential importance of MD simulations in revealing conformational shifts that static models might miss.

Comparative analysis of simulations of EphA2/A40s and EphA2/eA1 complexes supports the hypothesis that the aptamer mimics eA1’s binding mode, thus restoring EphA2 canonical signaling. Indeed, A40s and eA1 could share key EphA2 interacting residues, such as Asp53 and Arg103; however, the broader divergence in their binding profiles suggests opportunities for optimization. Residues uniquely engaged by eA1, like Gly49, Gln56, Asn57, Asp61, and His160, could guide the design of A40s variants with enhanced mimicry of eA1’s binding mode. For example, nucleotides 19–22, which are critical for binding within the EphA2 surface pocket, could be further modified to strengthen interactions with these specific residues. Such refinements might improve the aptamer’s capacity to activate EphA2’s canonical signaling, potentially unlocking therapeutic applications. It is, however, important to note that while specific nucleotides (e.g., positions 19–22) are critical for binding, the ability of A40s to recognize EphA2 cannot be solely attributed to sequence conservation. In this regard, we remark that sequence enrichment in SELEX may be influenced by factors beyond the binding interface. In particular, selective pressures imposed by the cellular environment, such as susceptibility to nuclease cleavage, off-target effects, or accessibility to processing machinery, may affect nucleotide conservation, thereby explaining the lack of a direct correlation between conservation scores and predicted structural interfaces.^93^ Structural modifications informed by our model could also improve the aptamer’s secondary structure stabilization, resistance to nuclease degradation, or enhance its pharmacokinetics without compromising its binding efficiency. Additionally, these insights can guide site-specific conjugation to enable multivalent binding. In recent years, multimeric aptamers targeting membrane receptors have been successfully developed, including a dimeric DNA aptamer that functions as a VEGFR2 agonist by promoting receptor dimerization,^11^ and two multivalent aptamers that act as CD28 agonists.^94^ Likewise, conjugated aptamers could be engineered to promote EphA2 clustering, potentially influencing receptor internalization, activation, or downstream signaling. This concept is supported by previous studies in which dimeric ligands on EphA2 led to higher-order EphA2 oligomers, increasing receptor tyrosine phosphorylation and downstream kinase-dependent signaling through two dimerization interfaces, whereas monomeric ligands dimerized via a single one.^95^ Therefore, our results not only shed light on the EphA2/A40s interface but also offer a foundation for the design of aptamers with improved biological activity.

Altogether, our study offers valuable hints for rational design strategies, including in silico screening of modified aptamer sequences before experimental validation. This computational approach is valuable for the development of optimized A40s variants, as it offers crucial insights into their interaction with EphA2. By rationally modifying this aptamer, we pave the way for more effective and specific therapeutic aptamers targeting EphA2 and related proteins while minimizing the need for extensive trial-and-error experiments, saving time and resources.

## Supplementary Material



## Data Availability

All the input files and trajectory data sets are published on a public Zenodo folder and freely available at the following link: 10.5281/zenodo.14870259.

## Notes

Clustering of A40s′ secondary and tertiary structures, the stability of A40s hairpin and bulge models as well as the stability and interactions of A40s and eA1 relative to EphA2 over MD simulations, semiquantitative analysis of RNase T1 cleavage at G19, and results concerning AlphaFold predictions (PDF)

## Abbreviations

AMD: age-related macular degeneration
CRD: cysteine-rich domain
FN3: fibronectin domain
eA1: ephrinA1
EphA2: erythropoietin-producing hepatocellular carcinoma A2
EGF: epidermal growth factor
EGFR: epidermal growth factor receptor
eRMSD: epsilon root mean square deviation
GA: geographic atrophy
GBM: glioblastoma multiforme
GSCs: glioblastoma stem cells
ILP: in-line probing
JM: juxtamembrane
LBD: ligand binding domain
MD: molecular dynamics
PBC: periodic boundary conditions
PDB: protein data bank
PME: particle-mesh Ewald summation method
RMSD: root-mean-square deviation
RMSF: root-mean-square fluctuations
RTK: receptor tyrosine kinase
SELEX: systematic evolution of ligand by exponential enrichment procedure
TanCAR: tandem CAR-T cell therapy
VEGF: vascular endothelial growth factor
VEGFR: vascular endothelial growth factor receptor
