# Supplementary material for: Targeting Glioblastoma Stem Cells via EphA2: Structural Insights into the RNA Aptamer A40s for Precision Therapy
Source: J Chem Inf Model. 2025 May 23;65(11):5635–48. doi: 10.1021/acs.jcim.5c00295 (PMC12152952; doi:10.1021/acs.jcim.5c00295)
Supplement: Supplementary file 1 [file ci5c00295_si_001.pdf]

SUPPORTING INFORMATION

Targeting Glioblastoma Stem Cells via EphA2: Structural Insights into the RNA Aptamer A40s for Precision Therapy

Isidora Diakogiannaki[1#], Vincenzo Maria D'Amore[1#], Alessandra Affinito[2#], Greta Donati[1], Elpidio Cinquegrana[1], Cristina Quintavalle[3], Martina Mascolo[2], Jule Walter[4], Heike Betat[4], Mario Mörl[4], Francesco Saverio Di Leva[1], Gerolama Condorelli[2*], Luciana Marinelli[1*]*

[1] Department of Pharmacy, University of Naples Federico II, Via Domenico Montesano 49, 80131 Naples, Italy

[2] Department of Molecular Medicine and Medical Biotechnology, University of Naples Federico II, Via Sergio Pansini 5, 80131 Naples, Italy

[3] Institute of Experimental Institute of Endotypes in Oncology, Metabolism and Immunology "G. Salvatore" (IEOMI), Consiglio Nazionale delle Ricerche (CNR), 80131 Naples, Italy

[4] Institute for Biochemistry, Leipzig University, Brüderstraße 34, 04103 Leipzig, Germany

[#] these authors equally contributed to the work

*corresponding authors

TABLE OF CONTENTS

Figure S1. Clustering of 2D structure predictions of A40s. S3

Table S1. Averaged RMSD values of A40s hairpin and bulged models over MD simulations. S4

Figure S2. Prediction of A40s 2D structure and of EphA2/A40s complex using AlphaFold. S4

Figure S3. eRMSD-based analysis of 3D A40s structure predictions. S5

Figure S4. Time evolution of the eRMSD over MD simulations of the A40s aptamer. S6

Figure S5. Cross eRMSD over MD simulations of the A40s aptamer. S7

Figure S6. Semi-quantitative analysis of RNase T1 cleavage at G19. S8

Figure S7. RMSD of A40s relative to EphA2 over the MD simulation on EphA2/A40s. S9

Figure S8. RMSD of A40s over the MD simulation on the EphA2/A40s complex. S9

Figure S9. DRPScore computed for the EphA2/A40s complex over MD timescale. S10

Figure S10. Histograms showing contacts over MD trajectories. S11

Figure S11. RMSD of eA1 over the MD simulations on the EphA2/eA1 complex. S12

References S13

Cluster 1

Centroid: ((((((((..............))))))))

Dot Bracket notation	Software	Method
((((((((..............))))))))	RNAstructure	Max Expect
((((((((..............))))))))	RNAfold	Centroid
(((((((((............)))))))))	CentroidFold	McCaskill (Turner)
(((((((((............)))))))))	Vfold2D	Turner (1st solution)
(((((((((............)))))))))	Vfold2D	Mfold (2nd solution)
((((((((..............))))))))	IPknot++	Linear Partition with ContraFold
((((((((..............))))))))	IPknot++	Linerar Partition with ViennaRNA
((((((((..............))))))))	IPknot++	McCaskill model with Boltzmann likehood
((((((((..............))))))))		McCaskill model with ViennaRNA
((((((((..............))))))))		CONTRAfold
((((((((..............))))))))		NUPACK
((((((((....(....)....))))))))	sFold	MFE (1st solution)
((((((((..............))))))))	KineFold	1st solution

Cluster 2

Centroid: ((((((((..((........))))))))))

Dot Bracket notation	Software	Method
((((((((..(((......)))))))))))	Vfold	Turner (2nd solution)
((((((((..(((......)))))))))))	Vfold	mFold (3rd solution)
((((((((..((........))))))))))	pkiss	MFE/Andronescu model 2007/no lonely pair (1st solution)
.(((((((..((........))))))))).	pkiss	MFE/Andronescu model 2007/no lonely pair (2nd solution)
((((((((..((........))))))))))	pkiss	MFE/Andronescu model 2007/allow lonely pair (1st solution)
.(((((((..((........))))))))).	pkiss	MFE/Andronescu model 2007/allow lonely pair (2nd solution)
((((((((..((........))))))))))	sFold	Centroid (2nd solution)
((((((((..(((.....).))))))))))	AlphaFold	A40s in complex with EphA2 (1st solution)
((((((((..((........))))))))))	AlphaFold	A40s in complex with EphA2 (2nd solution)
((((((((..((........))))))))))	AlphaFold	A40s in complex with EphA2 (3rd solution)

Cluster 3

Centroid: ((((((((..(.(....).)..))))))))

Dot Bracket notation	Software	Method
((((((((..(.(....).)..))))))))	CentroidFold	McCaskill (BL)
((((((((....(....)....))))))))	CentroidFold	CONTRAfold
((((((((..(.(....).)..))))))))	pkiss	MFE/Mathews model 2004/allow lonely pair
((((((((..(.(....).)..))))))))	sFold	MFE (2nd solution)
((((((((..(.........)..))))))))	sFold	Centroid (1st solution)
((((((((....(....)....))))))))	sFold	Centroid (3rd solution)
((((((((..(.(....).)..))))))))	AlphaFold	On A40s sequence (1st solution)
((((((((..(.(....).)..))))))))	AlphaFold	On A40s sequence (2nd solution)
((((((((..(.(....).)..))))))))	AlphaFold	On A40s sequence (3rd solution)

Cluster 4

Centroid: ((((((((((.....)).....))))))))

Dot Bracket notation	Software	Method
((((((((((.....)).....))))))))	RNAstructure	MFE
((((((((((.....)).....))))))))	RNAfold	MFE
((((((((((.....)).....))))))))	pkiss	MFE/Turner model 2004/no lonely pair
((((((((((.....)).....))))))))	pkiss	MFE/Turner model 2004/allow lonely pair
((((((((((.....)).....))))))))		MFE/Mathews model 2004/no lonely pair
((((((((((.....)).....))))))))	NUPACK	

Cluster 5

Centroid: (((((((((((((....)))))))))))))

Dot Bracket notation	Software	Method
(((((((((((((....)))))))))))))	MC-Fold	1st solution
((((((((((((((..))))))))))))))	MC-Fold	2nd solution
(((((((((.(((....))).)))))))))	MC-Fold	3rd solution

Cluster 6

Centroid: ((((((....)))))).............

Dot Bracket notation	Software	Method
((((((....))))))..............	sFold	MFE (3rd solution)
((((((....))))))..............	KineFold	2nd solution
((((((....)))..............)))	KineFold	3rd solution

Cluster 7

Centroid: ((((((((..((((...)).))))))))))

Dot Bracket notation	Software	Method
((((((((..((((...)).))))))))))	Vfold2D	Turner (3rd solution)
((((((((..((((...)).))))))))))	Vfold2D	Mfold (1st solution)

Figure S1. Clustering of 2D structure predictions of A40s using RNAstructure 6.5,[1] RNAfold 2.6.3,[2] CentroidFold 0.0.16,[3] NUPACK 4.0,[4] Vfold2D 2.5,[5] Ipknot++ 2.2.1,[6] MC–Fold 8,[7] KineFold 3,[8] pKiss 2.3.0,[9] sFold 2.2,[10] and AlphaFold 3.[11] All available parameter settings provided by each tool were considered for 2D structure prediction. For AlphaFold 3, which allowed for both isolated RNA and RNA-protein complex predictions, we included 2D structures of A40s predicted alone as well as those predicted in complex with EphA2. In cases where multiple structures with varying free energies were obtained, we selected the top three solutions, resulting in a total of 46 predicted 2D structures.

Table S1. Average values of RMSD and standard deviation of hairpin and bulged model of A40s calculated over the 3 µs MD simulations.

Replica ID	RMSD Hairpin (Å)	RMSD Bulged (Å)
**1**	2.4 ± 0.4	4.0 ± 1.0
**2**	2.7 ± 0.4	6.0 ± 1.6
**3**	3.3 ± 0.5	3.7 ± 0.8
**Average RMSD**	2.8 ± 0.4	4.6 ± 1.2

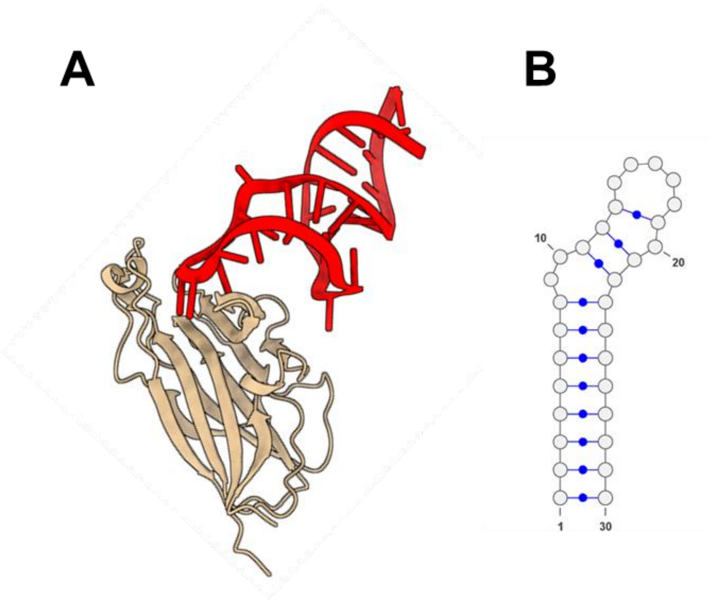

Figure S2. A) Prediction of EphA2/A40s complex using AlphaFold 3. B) 2D structure of A40s on EphA2 predicted by AlphaFold 3. The structure was not supported by the experimental data and was therefore excluded from further studies.

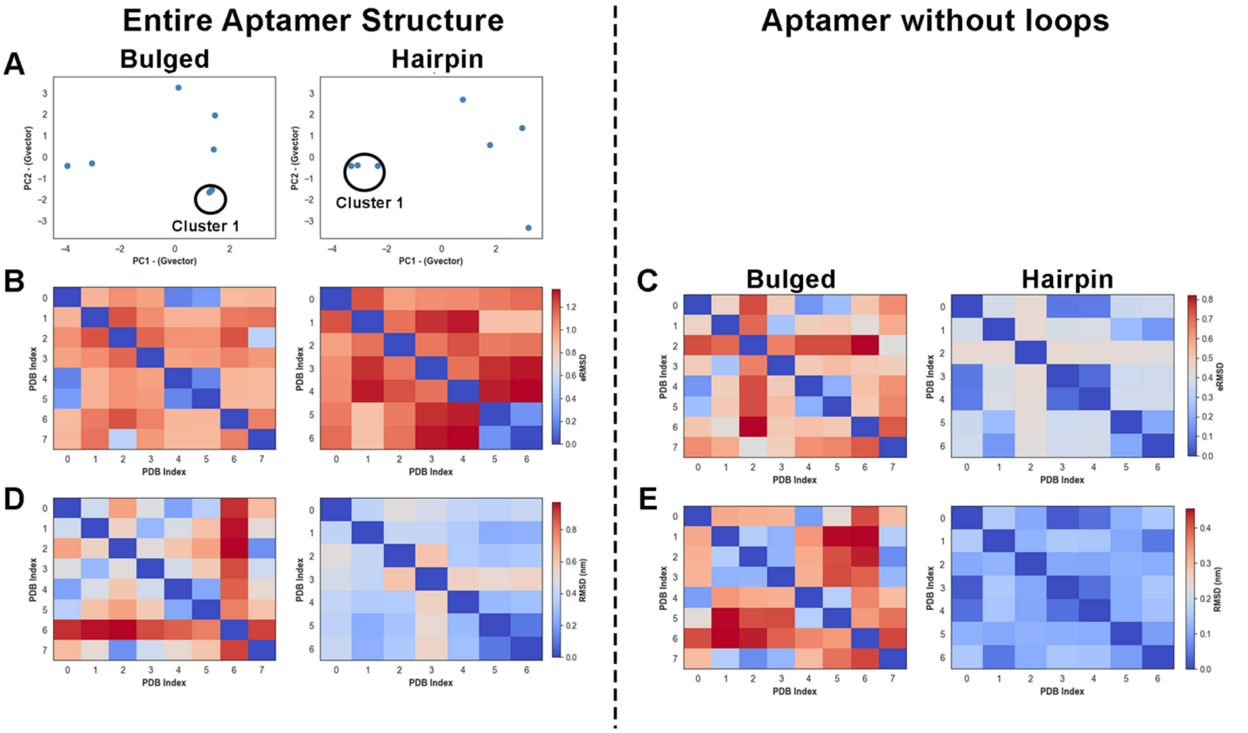

Figure S3. eRMSD-based analysis of 3D A40s structure predictions, alternatively including (A, B, D) or excluding (C, E) the aptamer's loop (nucleotides 9-22). A) Projection onto PC1 and PC2 of G-vectors computed from the PDBs provided by the 3D-conformation predictor tools for bulged (left) and hairpin (right) conformations. B) and C) Cross-eRMSD matrices between the PDBs generated by the 3D-conformation predictor tools for bulged (left) and hairpin (right) conformations. D) and E) Cross-RMSD matrices between the PDBs generated by the 3D-conformation predictor tools for bulged (left) and hairpin (right) conformations, based on the GROMOS algorithm as described in the manuscript.

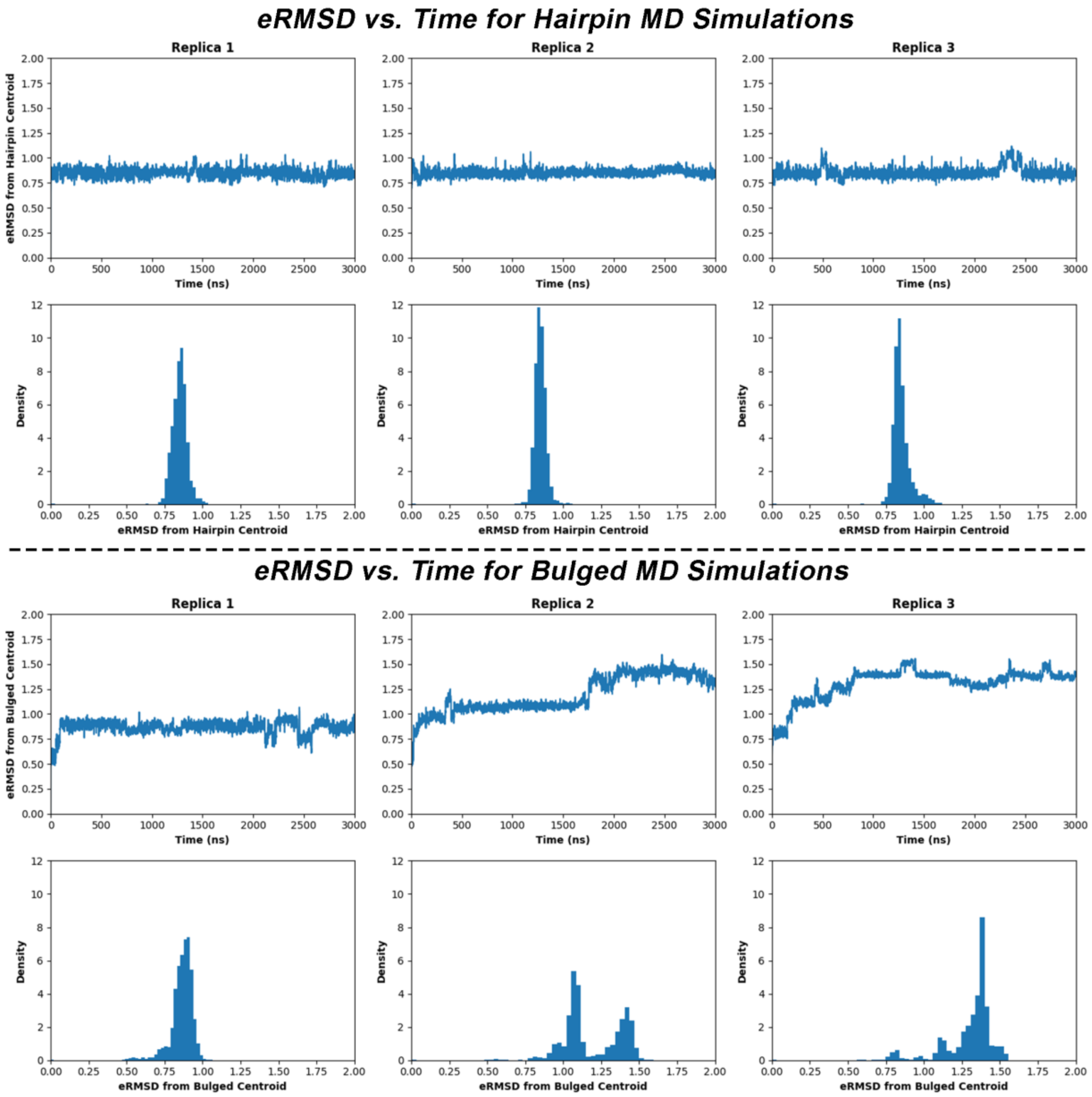

Figure S4. Time evolution of the eRMSD[12] and corresponding density histograms computed over three independent RNA-only MD simulations of the A40s aptamer, initiated from either the hairpin (upper panels) or bulged (lower panels) conformations. The initial offset in eRMSD values reflects the use of the respective cluster centroids (from hairpin or bulged ensembles) as reference structures for eRMSD calculation. These centroids undergo slight rearrangements during the initial equilibration phase, which are not included in the plotted trajectories.

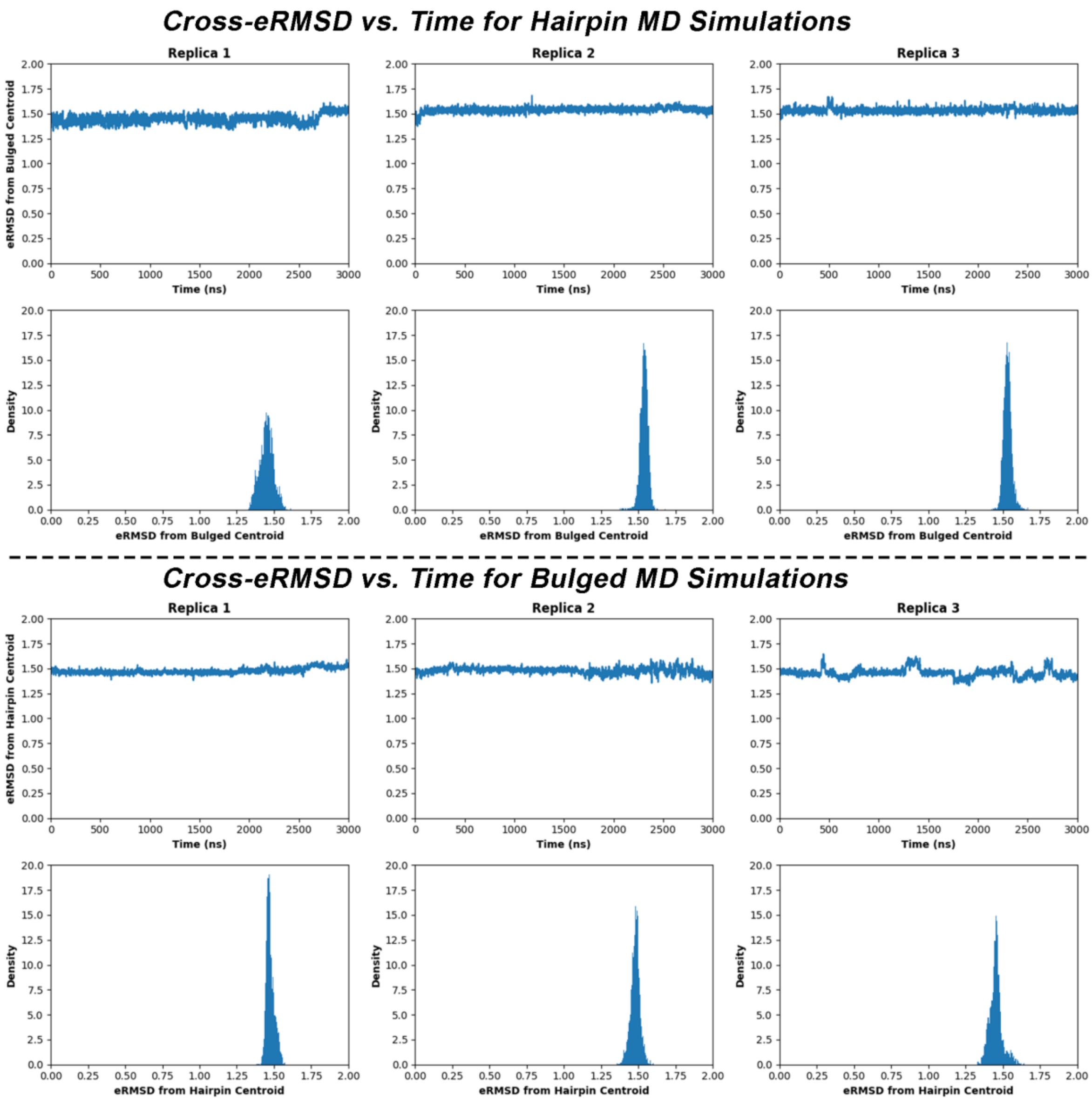

Figure S5. Cross-eRMSD evolution (upper row) and density histograms (lower row) computed for the A40s aptamer in three independent replicas of RNA-only simulations initialized from hairpin (upper panels) and bulged (lower panels) conformations. The reference conformations for eRMSD calculations are the centroids of the hairpin structure (when the simulation was initialized from the bulged conformation) and the bulged structure (when initialized from the hairpin conformation), both obtained through clustering.

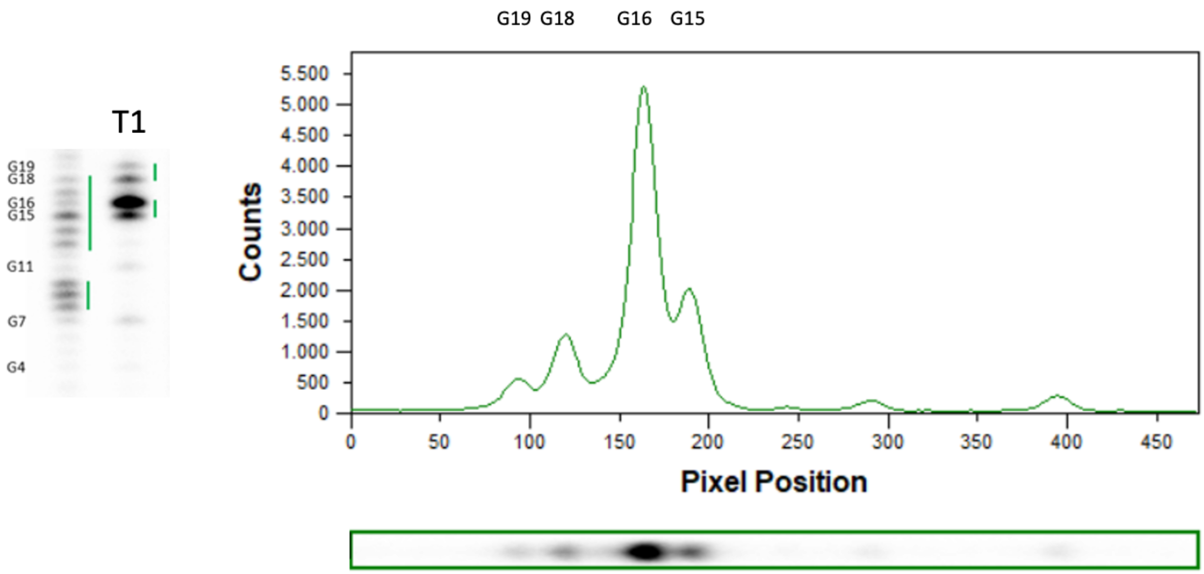

Figure S6. Semi-quantitative analysis of RNase T1 cleavage at G19. (Left) Autoradiograph showing cleavage at G residues, with G19 displaying markedly weaker cleavage compared to G15–G16. (Right) Densitometric profile of the gel, where peaks correspond to cleavage events. The weak signal at G19 supports transient unpairing at this position.

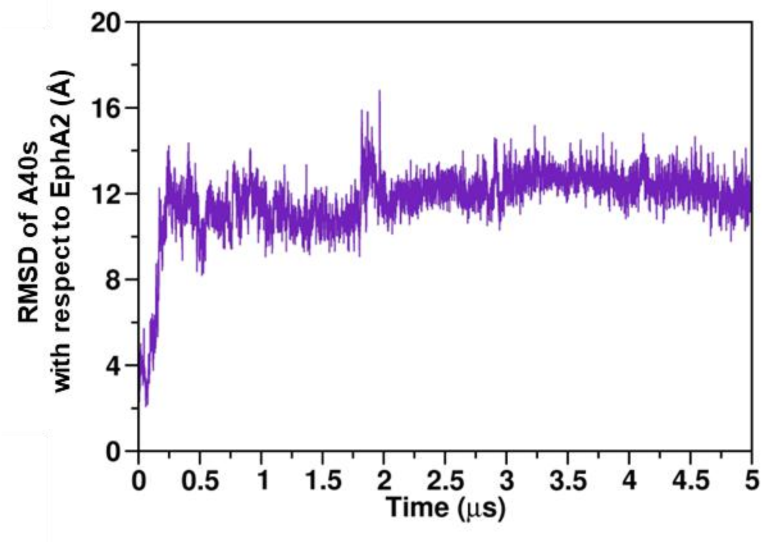

Figure S7. RMSD of A40s (calculated on heavy atoms, excluding terminal nucleotides) relative to EphA2 on the EphA2/aptamer complex. The trajectory was aligned on the EphA2 secondary structure Cα atoms.

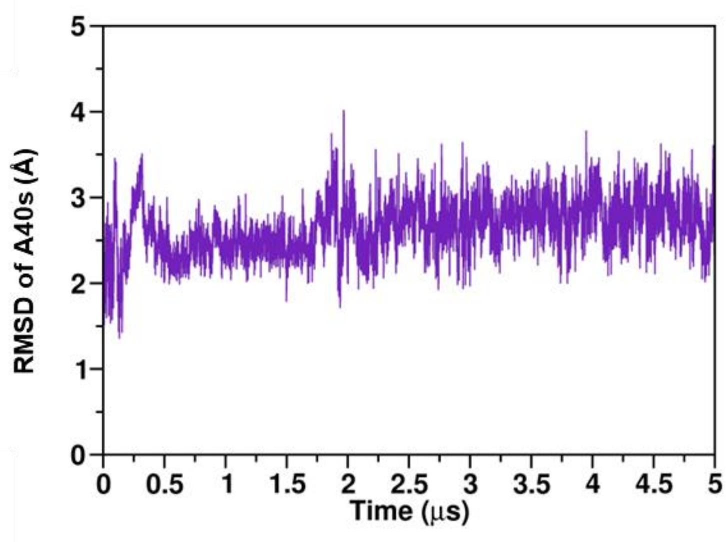

Figure S8. RMSD of A40s (calculated on heavy atoms, excluding terminal nucleotides) over the MD simulations on the EphA2/aptamer complex. The trajectory was aligned on the A40s heavy atoms, excluding the terminal nucleotides.

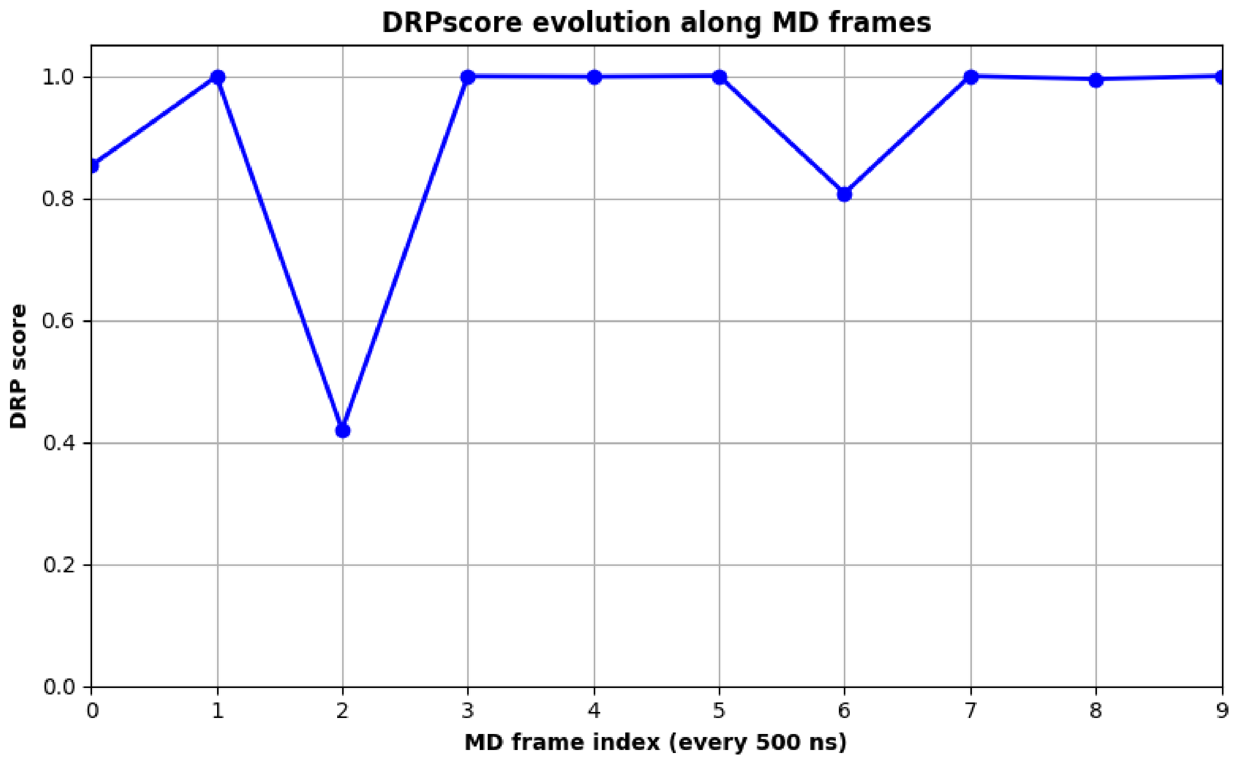

Figure S9. DRPScore[13] computed for the EphA2/A40s complex over MD timescale. Representative frames for DRPScore calculation were extracted each 500 ns.

A

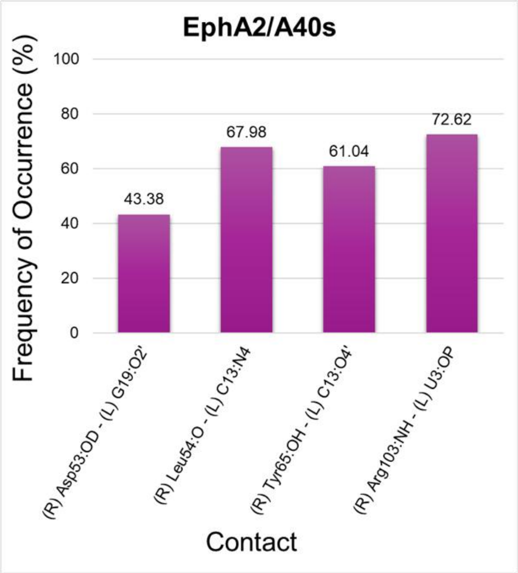

B

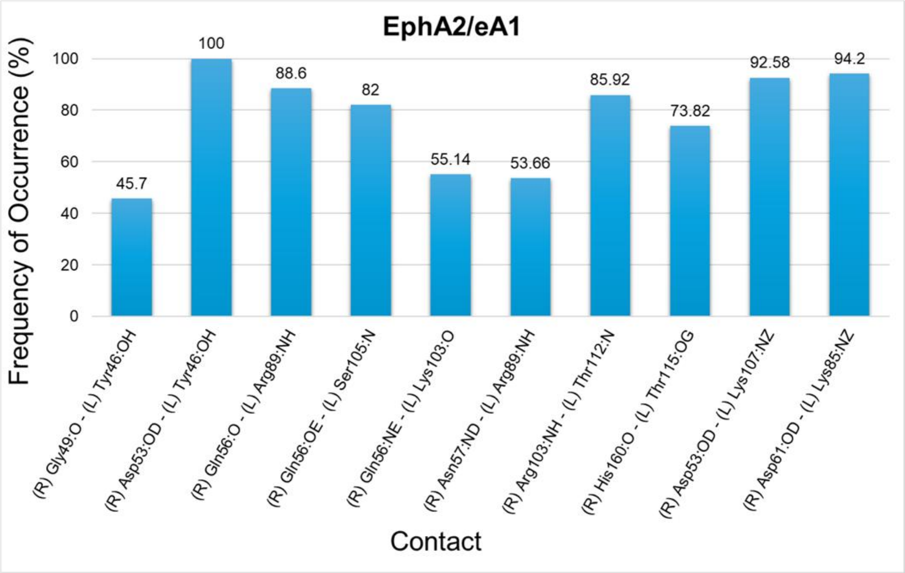

Figure S10. A) Histograms showing the frequency of occurrence of the most relevant EphA2/A40s contacts over the MD trajectory. B) Histograms showing the frequency of occurrence of the most relevant EphA2/eA1 contacts over the MD trajectory. For the identification of the non-covalent biomolecular interactions PyContact[14] was employed. The frequency of occurrence was obtained using the Hydrogen Bonds tool of VMD[15] where the donor-acceptor (heavy atoms) distance cutoff was set at 3.5 Å and the angle cutoff at 40°.

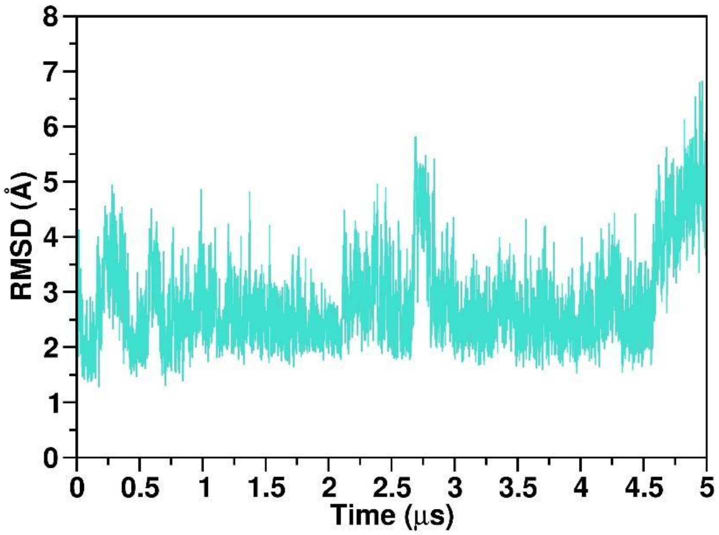

Figure S11. RMSD (calculated on the secondary Cα atoms) of eA1 over the MD simulations on the EphA2/eA1 complex. The trajectory was aligned on the secondary structure Cα atoms of EphA2.

References

1. Reuter, J. S.; Mathews, D. H. RNAstructure: software for RNA secondary structure prediction and analysis. *BMC Bioinformatics* **2010**, *11*, 129.
2. Lorenz, R.; Bernhart, S. H.; zu Siederdissen, C. H.; Tafer, H.; Flamm, C.; Stadler, P. F.; Hofacker, I. L. ViennaRNA Package 2.0. *Algorithms Mol. Biol*. **2011**, *6*, 26.
3. Sato, K.; Hamada, M.; Asai, K.; Mituyama, T. CENTROIDFOLD: A Web Server for RNA Secondary Structure Prediction. *Nucleic Acids Res.* **2009**, *37*, W277–W280. .
4. Zadeh, J. N.; Steenberg, C. D.; Bois, J. S.; Wolfe, B. R.; Pierce, M. B.; Khan, A. R.; Dirks, R. M.; Pierce, N. A. NUPACK: Analysis and Design of Nucleic Acid Systems. *J. Comput. Chem.* **2011**, *32*, 170–173.
5. Cheng, Y.; Zhang, S.; Xu, X.; Chen, S. J. Vfold2D-MC: A Physics-Based Hybrid Model for Predicting RNA Secondary Structure Folding. *J. Phys. Chem. B* **2021**, *125*, 10108–10118.
6. Sato, K.; Kato, Y.; Hamada, M.; Akutsu, T.; Asai, K. IPknot: Fast and Accurate Prediction of RNA Secondary Structures with Pseudoknots Using Integer Programming. *Bioinformatics* **2011**, *27*, i85–i93.
7. Parisien, M.; Major, F. The MC-Fold and MC-Sym Pipeline Infers RNA Structure from Sequence Data. *Nature* **2008**, *452*, 51–55.
8. Xayaphoummine, A.; Bucher, T.; Isambert, H. Kinefold Web Server for RNA/DNA Folding Path and Structure Prediction Including Pseudoknots and Knots. *Nucleic Acids Res.* **2005**, *33*, W605–W610.
9. Janssen, S.; Giegerich, R. The RNA Shapes Studio. *Bioinformatics* **2015**, *31*, 423–425.
10. Ding, Y.; Chan, C. Y.; Lawrence, C. E. Sfold Web Server for Statistical Folding and Rational Design of Nucleic Acids. *Nucleic Acids Res.* **2004**, *32*, W135–W141.
11. Abramson, J.; Adler, J.; Dunger, J.; et al. Accurate Structure Prediction of Biomolecular Interactions with AlphaFold 3. *Nature* **2024**, *630*, 493–500.
12. Bottaro, S.; Di Palma, F.; Bussi, G. The Role of Nucleobase Interactions in RNA Structure and Dynamics. *Nucleic Acids Res.* **2014**, *42*, 13306–13314.
13. Zeng, C.; Jian, Y.; Vosoughi, S.; Zeng, C.; Zhao, Y. Evaluating Native-like Structures of RNA-Protein Complexes through the Deep Learning Method. *Nat. Commun.* **2023**, *14*, 1060.
14. Scheurer, M.; Rodenkirch, P.; Siggel, M.; et al. PyContact: Rapid, Customizable, and Visual Analysis of Noncovalent Interactions in MD Simulations. *Biophys. J.* **2018**, *114*, 577–583.
15. Humphrey, W.; Dalke, A.; Schulten, K. VMD: Visual Molecular Dynamics. *J. Mol. Graph.* **1996**, *14*, 33–38.
